# An Efficient Two-Tier Causal Protocol for Mobile Distributed Systems

**DOI:** 10.1371/journal.pone.0059904

**Published:** 2013-04-09

**Authors:** Eduardo Lopez Dominguez, Saul E. Pomares Hernandez, Gustavo Rodriguez Gomez, Maria Auxilio Medina

**Affiliations:** 1 Research and Innovation Centre, Laboratorio Nacional de Informática Avanzada, Xalapa, Veracruz, Mexico; 2 Computer Science Department, Instituto Nacional de Astrofísica Óptica y Electrónica, Puebla, Mexico; 3 LAAS-CNRS, Tolouse, France; 4 Univ. de Toulouse, Tolouse, France; 5 Computer Science Department, Universidad Politécnica de Puebla, San Mateo, Puebla, Mexico; Cinvestav-Merida, Mexico

## Abstract

Causal ordering is a useful tool for mobile distributed systems (MDS) to reduce the non-determinism induced by three main aspects: host mobility, asynchronous execution, and unpredictable communication delays. Several causal protocols for MDS exist. Most of them, in order to reduce the overhead and the computational cost over wireless channels and mobile hosts (MH), ensure causal ordering at and according to the causal view of the Base Stations. Nevertheless, these protocols introduce certain disadvantage, such as unnecessary inhibition at the delivery of messages. In this paper, we present an efficient causal protocol for groupware that satisfies the MDS's constraints, avoiding unnecessary inhibitions and ensuring the causal delivery based on the view of the MHs. One interesting aspect of our protocol is that it dynamically adapts the causal information attached to each message based on the number of messages with immediate dependency relation, and this is not directly proportional to the number of MHs.

## Introduction

The deployment of mobile distributed systems (MDS), in conjunction with wireless communication technologies and Internet, enables portable computing devices (referred in this paper as *mobile hosts*), such as smart phones and personal digital assistants (PDAs), to communicate from anywhere and at anytime. The MDS deal with new characteristics and constraints such as host mobility implying changeable physical network connections, limited processing and storage capabilities in mobile hosts compared with desktop computers and limited bandwidth on wireless communication channels.

For mobile distributed systems, causal ordering algorithms are an essential tool to exchange information. The use of causal ordering provides built-in message synchronization and reduces the non-determinism induced by three main aspects: host mobility, asynchronous execution, and unpredictable communication delays. Causal ordering guarantees that actions, like requests or questions, are received before their corresponding reactions, results or responses. The concept of causal ordering has been of considerable interest in several domains, such as ubiquitous agents systems [1], context-aware systems [2] and multimedia synchronization protocols [3].

In this paper, we consider the problem of causal ordering message delivery among mobile hosts in the context of group communication. Recently, some protocols have been proposed to implement causal message ordering for mobile distributed systems [4], [5], [6], [7], [8], [9]. Nevertheless, most of these protocols, in order to reduce computational cost and communication loads on mobile hosts, ensure causal ordering at and according to the Base Stations (BSs). These methods give rise to two main problems. First, the causal order seen by the MHs (referenced in this paper as *mobile causal view*) greatly differs from the causal orders of messages in which they were originally sent. Secondly, these methods introduce unnecessary inhibition at the message delivery.

Other important aspect concerning the design of causal protocols for a MDS is mobility management. When a mobile host moves from a cell (source) to another cell (target), these protocols must continue to ensure the causal order of messages. To achieve this, they execute a handoff module. This module mainly consists on sending the main structures used between the source and target cells. However, most of them stop the sending of new messages among all mobile hosts during the execution of this procedure.

In this work, we propose a new protocol that ensures the causal ordering according to the causal view that the mobile hosts perceive in the MDS, avoiding the unnecessary inhibition at the message delivery, while maintaining a low overhead and computational cost. To achieve this, our causal protocol works at two communication levels according to the connection type: intra-base communication level and inter-base communication level. At the intra-base communication level (wireless connection), we only send as causal overhead timestamped per message, between a BS and the MHs, a structure of bits denoted by *h*(*m*). The *h*(*m*) is dynamically determined based on the immediate dependency relation, IDR [10]. In the best case, the size of *h*(*m*) is equal to 1 bit; and in the worst case, it is equal to *n* bits, (1≤|*h*(*m*)|≤*n*), where *n* is the number of *MHs* in the system.

At the inter-base communication level (wired connection) the causal overhead *H*(*m*) sent per message between BSs is composed of entries of the form (*p*, *t*), which are message identifiers, where *p* is the mobile host identifier, and *t* is the logical local clock of mobile host *p*. The size of the causal overhead *H*(*m*) is also dynamically determined by the IDR (1≤|*H*(*m*)|≤*n*). On the other hand, we propose a handoff management process that is characterized by allowing an asynchronous transfer of the mobile hosts among the cells of the system. This handoff management process does not interrupt the communication at any moment.

This paper is organized as follows. Section 2 presents the preliminaries (system model, background and definitions). The mobile causal protocol is provided in Section 3. In Section 4, we compare our protocol with the related works. Finally, conclusions are presented in Section 5.

## Materials and Methods

### The System Model

**Figure 1:**
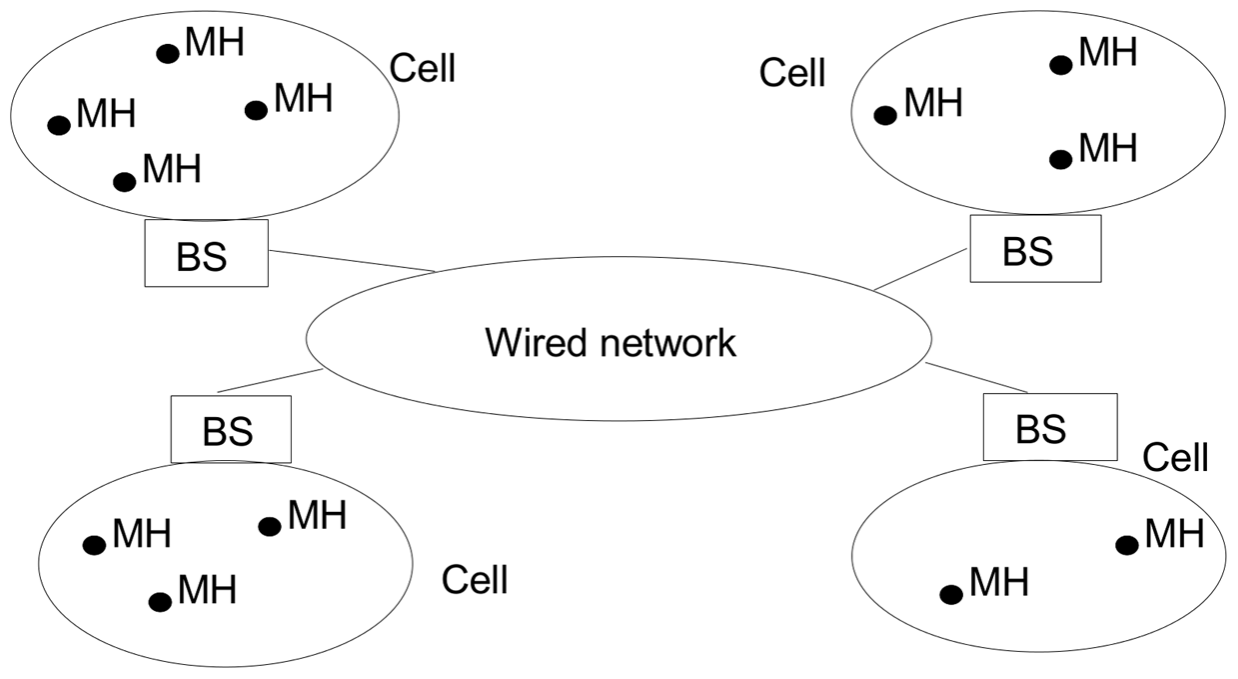
Physical architecture of a **MDS**.

We consider that a MDS runs over a wireless network infrastructure, which consists of two kinds of entities: base stations (BSs) and mobile hosts (MHs). A BS communicates with mobile hosts through wireless communication channels. The geographic area covered by a BS is called a cell, and it is depicted in Figure 1. At any given time, a MH is assumed to be within the cell of at most one BS, which is called its local BS. A MH can communicate with other MHs and BSs only through its local BS.

The base stations are connected among themselves using wired channels. The BSs and the wired channels constitute the static network. We assume that the wired channels are reliable, with an arbitrary but finite amount of time to deliver messages. Due to system asynchrony and unpredictable communication delays, the messages on a MDS from MH to MH can arrive in a different order as they were sent. In a mobile distributed system, a mobile host can move from one BS to another. In this case, a handoff procedure is performed to transfer the communication responsibilities of a MH to the new BS.

### Background and Definitions

Causal ordering delivery is based on the happened-before relation (HBR) defined by Lamport [11]. This relation establishes causal precedence dependencies over a set of events without using physical clocks. It is a partial order defined as follows:

#### Definition 1

The causal relation “→” is the least partial order relation on a set of events satisfying the following properties:

- If *a* and *b* are events belonging to the same process, and *a* was originated before *b*, then 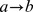.
- If *a* is the sent message of a process, and *b* is the reception of the same message in another process, then 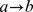.
- If 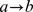 and 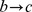, then 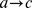.

By using Definition 1, we say that a pair of events is concurrently related “
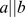
” only if 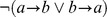.

The precedence relation on messages denoted by 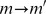 is induced by the precedence relation of events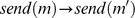.

#### The Immediate Dependency Relation

The Immediate Dependency Relation (IDR) [10] is the transitive reduction of the HBR. We denote it by ↓, and it is defined as follows:

#### Definition 2

Immediate Dependency Relation “↓” (IDR):
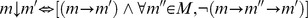



Thus, a message *m* directly precedes a message *m*', if and only if no other message *m*" belonging to *M* exists (*M* is the set of messages of the system), such that *m*" belongs at the same time to the causal future of *m* and to the causal past of *m*'.

#### Broadcast Causal Delivery

The causal delivery for group communication (broadcast case) based on the IDR is defined as follows:
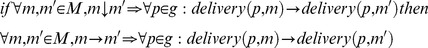



## Results

### Protocol composition

**Figure 2:**
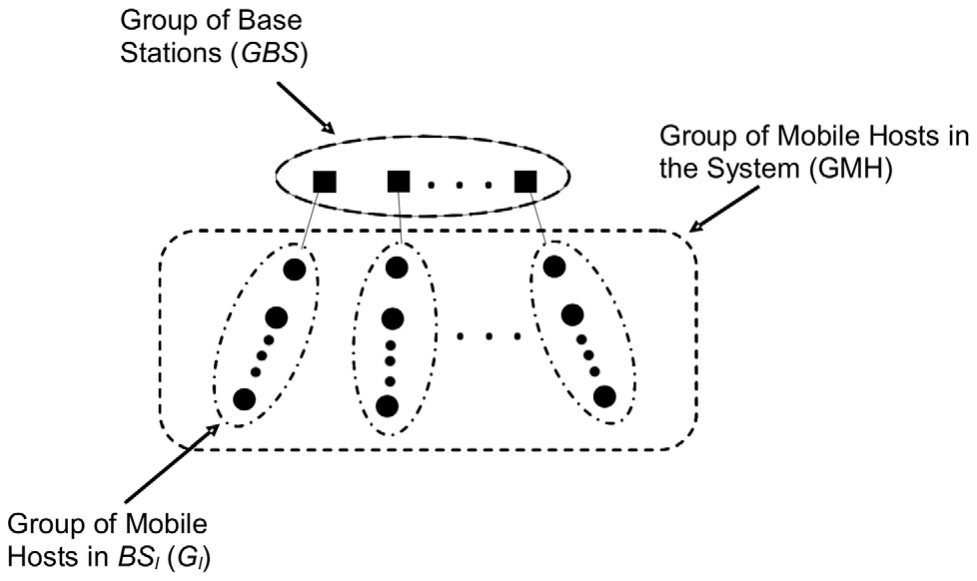
Logical structure considered for a mobile distributed system.

From a logical point of view, we consider that the entities of the MDS are structured into two main communication groups, one conformed by the base stations (*GBS* = {*BS*_1_, *BS*_2_,*…, BS_s_*}), and the other integrated by mobile hosts (*GMH* = {*p*_1_, *p*_2_,*…, p_n_*}), where *s* and *n* are the number of base stations and mobile hosts, respectively. The *GMH* is subdivided into subgroups (*G_l_*); one for each *BS* (see Figure 2).

The *BSs* in the *GBS* and the mobile hosts in a *G_l_* communicate by reliable asynchronous message passing. We consider a finite set of messages *M* with *m*ϵ*M*, identified by a tuple *m* =  (*p*, *t*), where *p* is the sender mobile host, such that *p*ϵ*GMH* and *t* is the logical clock for messages of *p* when *m* is sent. When we need to refer to a specific process with its respective identifier, we write *p_i_*. The set of destinations of a message *m* is always a *GMH*.

In our work, the *GBS* carries out the causal delivery of messages according to the order in which messages were observed by the mobile hosts. To achieve this, each mobile host *p* uses a structure of bits *Φ*(*p*) in order to establish an immediate dependency relation (Definition 2) among messages. The content of *Φ*(*p*) is the only control information attached per message in the wireless channel (1≤|*Φ*(*p*)|≤*n*). Each bit in *Φ*(*p*) identifies a causal message *m* that has a potential IDR with the next message to be sent by *p*. The base stations keep the main structures of causal control information of the mobile distributed system, such as the vector clock *VT* introduced by Mattern [12]. Through the control information and the structure of bits *h*(*m*)←*Φ*(*p*) sent in the system, the base stations can determine the immediate dependency relation among the messages sent by *MHs* on different *BSs*.

### Data Structures

Each **mobile host** *p* uses and stores the following data structures:

- *mes_received*(*p*) is a counter, which is incremented each time a message is received by the mobile host *p*.
- *mes_sent*(*p*) is a counter, which is incremented each time a message is sent by the mobile host *p*.
- *Φ*(*p*) is a structure of bits. Each bit in *Φ*(*p*) identifies a message *m* in the causal past of *p* that has a potential *IDR* with the next message to be sent by *p*. The size of *Φ*(*p*) fluctuates between 1≤|*Φ*(*p*)|≤*n*.

Each **base station** *BS* uses and stores the following data structures:

- *mes_sent*(*BS*) is a counter which is incremented each time a message is sent by the base station *BS* in its cell.
- *VT*(*BS*) is the vector clock of Mattern [12]. For each mobile host *p*, there is an element *VT*(*BS*)[*i*] where *i* is the mobile host identifier of *p_i_*.
- *CI*(*BS*) is a control information structure. It is a set of entries (*i, t, d, ip*). The entry (*i, t, d, ip*) represents a message sent by the mobile host *p_i_* with logical local clock *t* = *VT*(*BS*)[*i*] and *d = mes_sent*(*BS*). Finally, *ip* is a Boolean variable. The *BS* sets *ip* to *true* when it detects that a recently message received has an immediately dependency relation with the message *m* = (*i, t, d, ip*). In Section protocol description we present a detailed description of how this procedure is carried out.

#### Message structures

**Figure 3:**
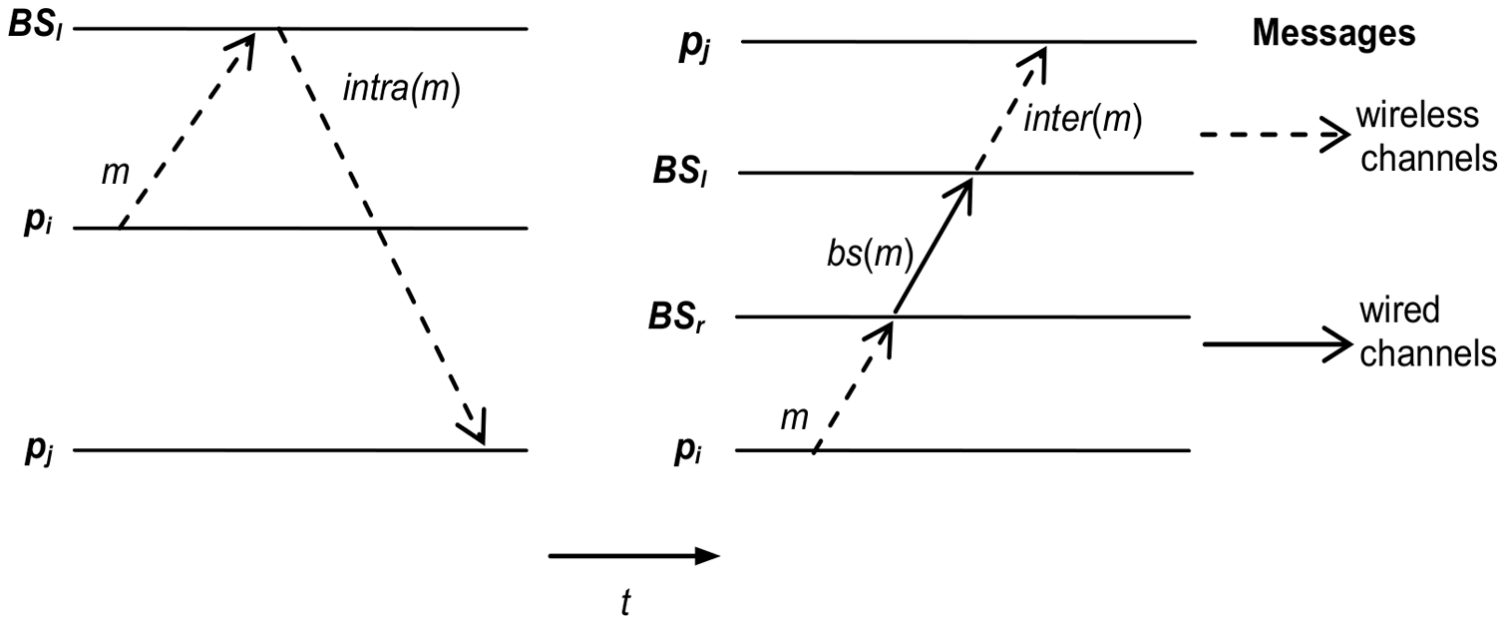
Messages sent at the intra-base and inter-base levels, respectively.

The following message structures are used in the *MDS* by the mobile hosts and base stations (see Figure 3).

- The messages sent in the wireless communication channels by mobile hosts to their base station are identified by *m*, and have the following form: *m≡*(*i*, *t*, *mes_received*(*p*), *data, h*(*m*)), where the structures *i*, *t*, and *mes_received*(*p*) have been previously described and:
  1. *h*(*m*) is a structure of bits. Each bit in *h*(*m*) identifies a message in the causal past of *p_i_* that has IDR with *m*.
- A message *m* sent among base stations BSs is denoted by *bs*(*m*), and it is composed by a quadruplets *bs*(*m*) *≡* (*i, t*, *data, H*(*m*)), where the structures *i*, *t* have been previously described and:
  1. *data* is the content of the message, and
  2. *H*(*m*) is composed of a set of elements (*i*, *t*), which represent messages that have an IDR with *m*.
- A message *m* received by a *BS_l_* from a mobile host *p*ϵ*G_l_* and which has been resent by such *BS_l_* in its cell, consists of a quintuplet that we call *intra*(*m*) *≡* (*i, t*, *data, h*'(*m*)).
  1. *h*'(*m*) is a structure of bits. Each bit in *h*'(*m*) identifies a message in the causal past of *p_i_* that has an IDR with *m* and that the *BS_l_* has not ensured its causal delivery.
- A message *bs*(*m*) received by a *BS_l_* and which has been resent within its cell, consists of a quintuplet that we call *inter*(*m*) *≡* (*i, t*, *data, h*'(*m*)).

### Specification of the MOKA Protocol

Structures and variables at mobile host *p_i_* are initialized as follows:

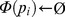

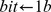

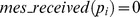

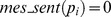

Structures and variables at *BS_r_* are initialized as follows:

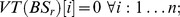

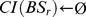

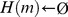

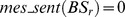

Next, we present in Tables 1–4 our causal protocol for groupware which satisfies the MDS's constraints, avoiding unnecessary inhibitions and ensuring the causal delivery based on the view of the MHs.

**Table 1:** Diffusion of message *m* by a mobile host *p_i_*_._

	For each diffusion of message send(*m*) at mobile host *p_i_*
1.	*mes_sent*(*p_i_*) = *mes_sent*(*p_i_*) +1
2.	*h*(*m*) ← *Φ*(*p_i_*)
3.	*m* ** = **(*i,t = mes_sent*(*p_i_*),*mes_received*(*p_i_*),*data,h*(*m*))
4.	**Diffusion:** send(*m*) /* sent of message *m* to local BS*/
5.	*Φ*(*p_i_*) ← Ø
6.	*mes_received*(*p_i_*) = *mes_received*(*p_i_*) + 1

**Table 2:** Reception of message *intra(m)* or *inter(m)* by a mobile host *p_j,_ i*≠*j*.

	/* *intra*(*m*) * = * (*i, t*, *data, h*'(*m*)) or *inter*(*m*) * = * (*i, t*, *data, h*'(*m*)) */
1.	**if** not (*t* = *mes_received*(*p_j_*) + 1) then
2.	*wait* (*intra*(*m*) *| inter*(*m*))
3.	**Else**
4.	*delivery*(*intra*(*m*) *| inter*(*m*))
5.	*mes_received*(*p_j_*) = *mes_received*(*p_j_*) + 1
6.	µ {*bit*} ∈ *h*'(*m*)
7.	*Φ*(*p_j_*) ← *Φ*(*p_j_*)/{*bit*}
8.	*Φ*(*p_j_*) ← *Φ*(*p_j_*) ∪ {*bit*} **endif**

**Table 3:** Reception of message *m = (i,t,mes_received(p_i_),data,h(m))* and sending of *intra(m)* by a base station *BS*.

1.	**if** *i* ∈ *BS_r_* then
2.	** if** not (*t* = *VT*(*BS_r_*)[*i*] *+*1) then
3.	* wait*(*m*)
4.	** Else**
5.	* delivery*(*m*)
6.	* VT*(*BS_r_*)[*i*] = *VT*(*BS_r_*)[*i*] +1
7.	µ {*bit*} ∈ *h*(*m*)
8.	** if** ∃ (*k*,*t*,*d,ip*) *∈ CI*(*BS_r_*) | *d* = *mes_received*(*p_i_*) and *ip* = *false* then
9.	* h*'(*m*) ← *h*'(*m*) ∪ {*bit*}
10.	(*k*, *t*, *d*, *ip*) ← (*k*, *t*, *d, ip = true*) **endif**
11.	** if** ∃(*k*,*t*,*d*,*ip*)*∈ CI*(*BS_r_*) | *d* = *mes_received*(*p_i_*) then
12.	* H*(*m*) ← *H*(*m*) ∪ (*k*, *t*) **endif**
13.	* mes_received*(*p_i_*) * = mes_received*(*p_i_*) – 1
14.	* intra*(*m*) * = * (*i, t = mes_sent*(*BS_r_*) *+* 1, *data, h*'(*m*))
15.	* bs*(*m*) * = * (*i, t*, *data, H*(*m*))
16.	** Diffusion:** *send*(*intra*(*m*)) /* sending of message *intra*(*m*) to local mobile hosts */
17.	** Diffusion:** *send*(*bs(m)*) /* sending of message *bs(m)* to the other bases stations */
18.	**endif**
19.	*mes_sent*(*BS_r_*) * = mes_sent*(*BS_r_*) *+* 1
20.	*CI*(*BS_r_*) ←*CI*(*BS_r_*) ∪ { (*i*,*t,mes_sent*(*BS_r_*), *ip = false*) } **endif**

**Table 4:** Reception of message *bs*(*m*) *≡* (*i*, *t*, *data*, *H*(*m*)) and sending of *inter*(*m*) by a base station *BS_r_*, such that *i* ∉ *BS_r_*.

1.	**if** *i* ∉ *BS_r_* then
2.	**if** not (**(** *t* = *VT*(*BS_r_*)[*i*]*+*1**)** and **(**µ (*s,x*)∈ *H*(*m*): *x* ≤ *VT*(*BS_r_*)[*s*]**))** then
3.	* wait*(*bs*(*m*))
4.	**else**
5.	* delivery*(*bs*(*m*))
6.	* VT*(*BS_r_*)[*i*] = *VT*(*BS_r_*)[*i*] +1
7.	µ (*x,y*) ∈ *H*(*m*)
8.	** if** ∃ (*k*,*t*,*d, ip*) *∈ CI*(*BS_r_*) | *x* = *k* and *y* = *t* and *ip = false* then
9.	* h*'(*m*) ← *h*'(*m*) ∪ {*bit*}
10.	(*k*,*t*,*d*, *ip*) ← (*k*,*t*,*d*, *ip = true*) **endif**
11.	* inter*(*m*)* = * (*i, t = mes_sent*(*BS_r_*) *+* 1, *data, h*'(*m*))
12.	** Diffusion:** send(*inter*(*m*)) /* sending of message *inter*(*m*) to local mobile devices */
13.	** endif**
14.	**endif**
15.	*mes_sent*(*BS_r_*) * = mes_sent*(*BS_r_*) *+* 1
16.	*CI*(*BS_l_*) ←*CI*(*BS_r_*) ∪ { (*i*,*t,mes_sent*(*BS_r_*), *ip = fase*) }

### Protocol Description

The main contribution of the present paper is to ensure causal ordering according to the causal view at the *GMH*. In this section, we focus on how our protocol performs that causal ordering at the wireless network level. A description of the causal ordering algorithm for the wired network i.e. the group of base stations level is presented in [10].

**Figure 4:**
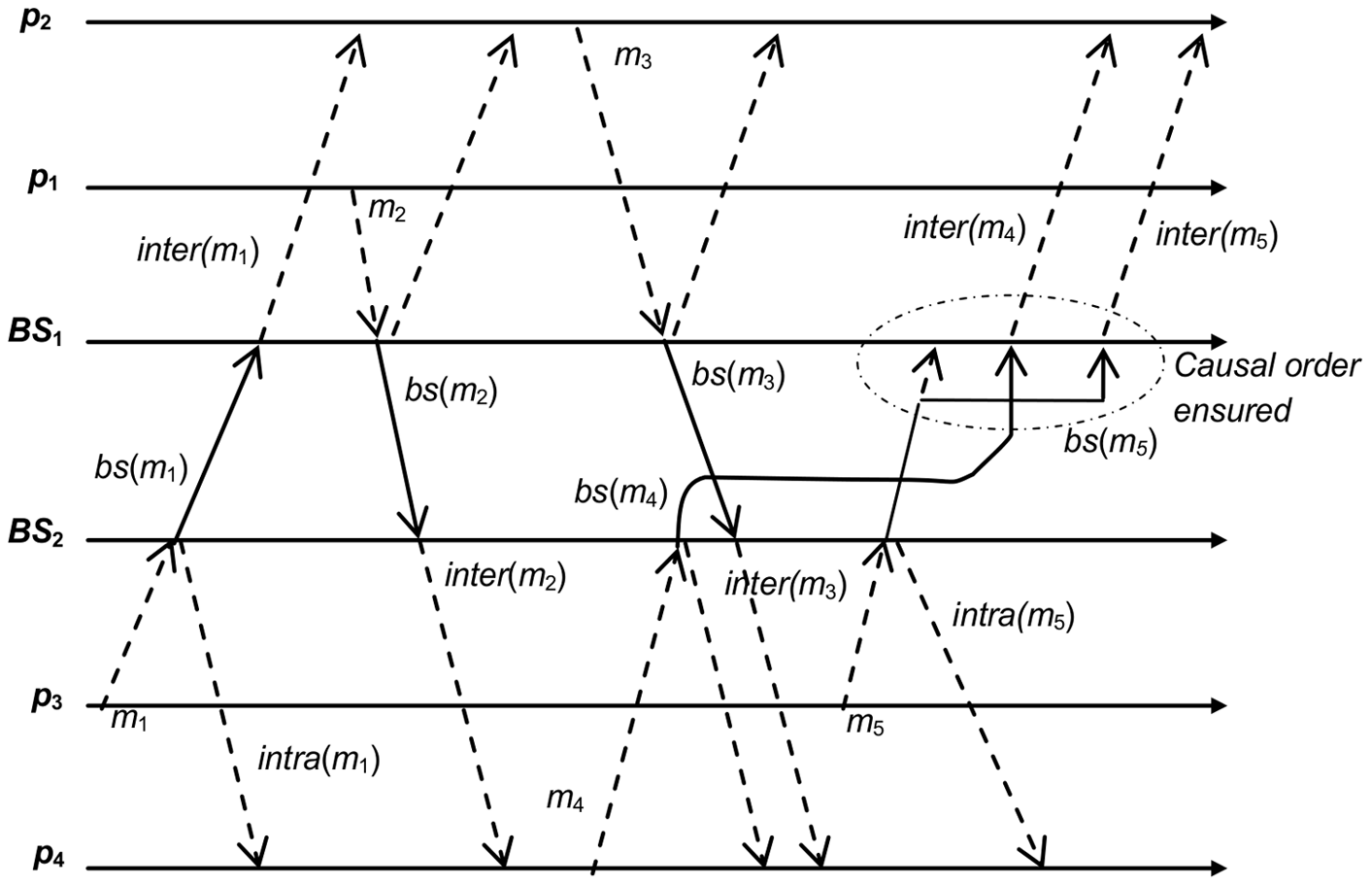
Scenario of communication group composed by four mobile hosts and two *BS*s.

In this Section we will refer to Figure 4 in order to explain how our protocol ensures causal ordering. In this scenario, the group of mobile hosts is composed by *GMH*  =  {*p*_1_, *p*_2_, *p*_3_, *p*_4_} and the group of base stations is integrated by *GBS*  =  {*BS*_1_, *BS*_2_} where *p*_1_, *p*_2_ ϵ *BS*_1_ and *p*_3_, *p*_4_ ϵ *BS*_2_. In order to show how our protocol ensures the causal order, we focus on the message *m*_5_ sent by *p*_3_ and its delivery to the mobile hosts at *BS*_1_.

Prior to the delivery of *m*_5_ to *BS*_2_, the control information at *BS*_1_ and *BS*_2_ are: *CI*(*BS*_1_)  =  ({*p*_1_, 1, 2, *true*}, {*p*_2_, 1, 3, *false*}), *CI*(*BS*_2_)  =  ({*p*_1_, 1, 2, *true*}, {*p*4, 1, 3, *false*}, {*p*_2_, 1, 4, *false*}), *VT*(*BS*_1_)  =  (1, 1, 1, 0) and *VT*(*BS*_2_)  =  (1, 1, 1, 1). These values are deduced from our protocol shown in Tables 1–4; see Section specification of the Moka protocol. According to our algorithm, the delivery process is described as follows.

At the diffusion of message *m*_5_ by *p*_3_ to *BS*_2_, **Table 1** **, Lines 1–6** (henceforth, we will use “Lines 1, 1–6” to simplify), the value of *mes_sent*(*p_i_*) is increased by one, *mes_sent*(*p_i_*)  = 2, **(Line 1, 1).** The mobile host *p*_3_ copies the bits structure *Φ*(*p*_3_) to *h*(*m*_5_), **(Line 1, 2)**. Message *m*_5_ *= *(*p*_3_, 2, 4, *data*, *h*(*m*_5_)  = 11) is constructed and sent in **(Line 1, 3–4)**. Through *h*(*m*_5_)  = 11 local *BS*_2_ will be able to determine which messages immediately precede *m*_5_.

When message *m*_5_ is received at *BS*_2,_ the FIFO delivery condition is verified **(Line 3, 2)**. From our scenario, this condition is satisfied. Then the message *m*_5_ is delivered to *BS*_2_ and the *VT*(*BS*_2_) vector is increased by one at the position *p*3, *VT*(*BS*_2_) = (1, 1, 2, 1). Later on, the *BS*_2_ sends message *m*_5_ to *BS*_1_. This is done through the diffusion of message *bs*(*m_5_*) by *BS*_2_.

The message *bs*(*m_5_*) is constructed by *BS*_2_ as follows. According to *h*(*m*_5_) structure, there are two messages that immediately precede *m*_5_. In order to identify these messages, **(Lines 3, 7–15)**, *BS*_2_ determines if there are some elements in *CI*(*BS*_2_) with *d* equal to variable *mes_received*(*p*_3_) of *m*_5_. In this case, there is an element at *CI*(*BS*_2_) related to *p*_2_ with *d* = 4 **(Line 3, 11)**. Afterwards, the variable *mes_received*(*p*_3_) is decremented by one **(Line 3, 13).** In the next iteration of the algorithm, *BS*_2_ found another element at *CI*(*BS*_2_) with respect to *p*_4_ with *d* = 3 **(Line 3, 11)**. Therefore, the only control information attached to *bs*(*m*_5_) in order to ensure a causal order relates to *m*_3_ and *m*_4_, which are the only messages that have an immediate dependency relation with *bs*(*m*_5_), see Figure 4. Hence, the message sent from *BS*_2_ to *BS*_1_ is *bs*(*m*_5_)* = *(*p*_3_, 2, *data*, *H*(*m*_5_) = ({*p*_2_,1}, {*p*_4_,1})) **(Line 3, 17)**.

When message *bs*(*m*_5_) is received at base station *BS*_1_, see Figure 4, *BS*_1_ verifies that the message satisfies the FIFO and causal delivery condition, **(Line 4, 2)**. In this case, message *bs*(*m*_5_) satisfies only the FIFO delivery condition **(Line 4, 2)** because *t* = 2 and *VT*(*BS*_1_)[*p*_3_] = 1, (*t* =  *VT*(*BS*_1_)[*p*3]+1). Due to the fact that message *m*_4_ has not been received by mobile hosts within the cell of *BS*_1_, the causal delivery condition (1≤*VT*(*BS*_1_)[*p*_4_] = 0) is not satisfied; therefore the message *bs*(*m*_5_) cannot be delivered causally and it is delayed **(Line 4, 3)**.

According to this scenario, message *bs*(*m*_4_) is received by *BS*_1_ **, (Lines 4, 1–14).** *BS*_1_ verifies that message *bs*(*m*_4_) satisfies the FIFO and causal delivery condition. In this case, *bs*(*m*_4_)* = *(*p*4, 1, *BS*_2_, *data*, *H*(*m*_4_) = (*p*_1_,1)) satisfies both conditions **(Lines 4, 2)**. Therefore, message *bs*(*m*_4_) is delivered, and the vector is increased by one in *VT*(*BS*_1_)[*p*_4_] resulting in *VT*(*BS*_1_) = (1, 1, 1, 1). Later on, *BS*_1_ sends the message *bs*(*m*_4_) to its local mobile hosts. The message to be sent by *BS*_1_ to local mobile hosts is *inter*(*m*_4_) * = * (*p*_4_, 4, *data*, *h*'(*m*_4_)  = 0), **(Lines 4, 12)**.

The delivery of message *inter*(*m*_4_) by mobile host *p*_1_ is as follows, **(Lines 2, 1–8)**. The mobile host *p*_1_ updates *Φ*(*p*_1_) with the attached information of message *inter*(*m*_4_) **(Lines 2, 6–8)**. The structure of bits after updating the data structures at *p*_1_ is *Φ*(*p*_1_) = 11. Afterwards, the variable *mes_received*(*p*_1_) is increased by one, *mes_received*(*p*_1_) = 4.

Finally, after the delivery of message *bs*(*m*_4_) to mobile host *p_1_*, *BS*_1_ verifies if message *bs*(*m*_5_) satisfies the causal delivery condition **(Line 4, 2)**. Now, message *bs*(*m*_5_) satisfies the causal delivery condition, 1≤*VT*(*BS*_1_)[*p*4] = 1, because of message *m*_4_ has been received by mobile hosts within the cell covered by *BS*_1_. Therefore, the message *bs*(*m*_5_) can be delivered causally. *BS*_1_ must send the message *bs*(*m*_5_) to its local mobile hosts. The message sent by *BS*_1_ to local mobile hosts is *inter*(*m*_5_)* = *(*p*3, 5, *data*, 11), **(Line 4, 12)**. When message *inter*(*m*_5_) is received by mobile host *p*_1_, **(Lines 2, 1–8)**, its delivery is done in the same way as it was previously described for *inter*(*m*_4_).

### Handoff Management Process

**Figure 5:**
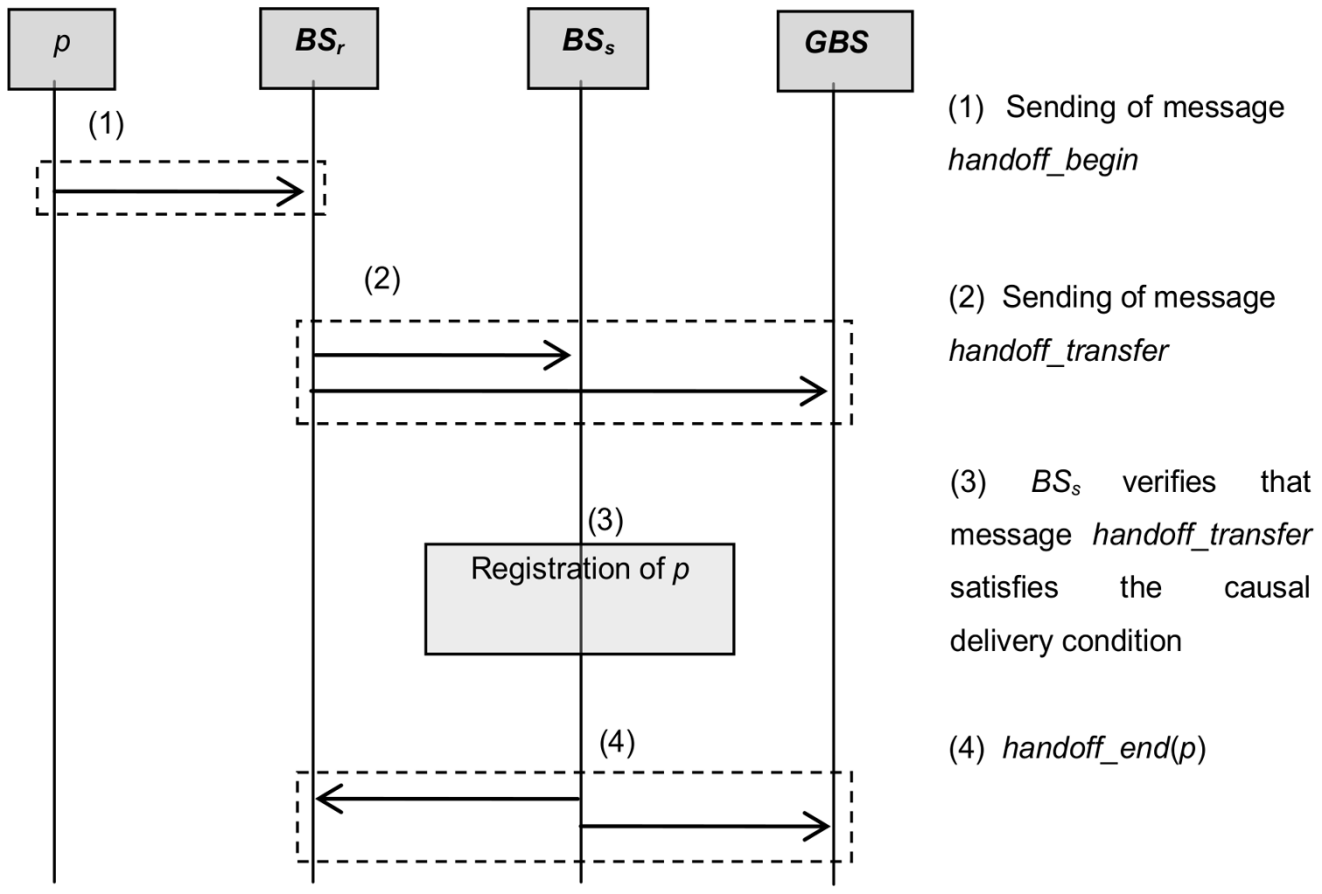
Description of the handoff module.

When a mobile host *p* in a cell covered by *BS_r_* moves to a cell covered by *BS_s_*, the responsibility of maintaining its causal dependencies shifts from the base station *BS_r_* to *BS_s_*. In order to ensure a causal ordering of messages in a mobile distributed system, the handoff module described in this Section is executed. In our case, we send only a control message with causal information about *p* in order to ensure a causal ordering of messages at the group of mobile hosts (*GMH*). The steps carried out by the handoff management process are depicted in Figure 5.

Assume that a mobile host *p* located in the cell covered by the source base station *BS_r_* moves to a cell covered by the base station *BS_s_* (see Figure 5). The first step established by *p* is to send the message *handoff_begin = *(*p*, *t*, *BSs*, *h*(*handoff_begin*) = *Φ*(*p*)) to its *BS_r_* (Figure 5). Upon receiving this message, *BSr* informs *BSs* and other base stations in the *GBS* that *p* is switching from base station *BS_r_* to *BS_s_* by sending the message *handoff_transfer = *(*p, t, H*(*handoff_begin*)), where *H*(*handoff_begin*) is the causal history of mobile host *p*, see Figure 5. The structure *H*(*handoff_begin*) contains the identifiers of the last messages received by *p*, when it was over the cell covered by *BS_r_*.

In order to ensure the causal order, when the target *BS_s_* receives the message *handoff_transfer*  =  (*p, t, H*(*handoff_begin*), it verifies that this message satisfies the causal delivery condition. If the message satisfies the causal delivery condition, then it is delivered and *BS_s_* makes the registration of *p* as a new mobile host in its cell, see Figure 5. Otherwise, the message is delayed until the causal condition is satisfied. The ending of the handoff procedure is identified by the diffusion of message *handoff_end*(*p*) by *BS_s_* to the other base stations, see Figure 5. After this message, *BSs* takes care of maintaining the causal ordering of messages sent to *p*.

In our handoff management process, we attach causal information about *p* to message *handoff_transfer* sent by the *BS_r_*. This causal information is used by the target base station (*BS_s_*) in order to determine if the mobile host served by *BS_s_* has received the messages observed by the mobile host *p* when it was over the cell covered by the source base station (*BS_r_*). If the message *handoff_transfer* satisfies the causal delivery condition, the mobile host *p* can be registered by the target base station because the messages observed by *p* have been received by the mobile hosts served by *BS_s_*. Otherwise, the mobile host *p* cannot be registered by the target base station because the messages observed by mobile host *p* when it was in the source base station can be received again by it, violating the causal order.

In our case, we do not attach data structures, such as vector clock (*VT*(*BS*)), to the messages sent during the handoff management process in order to maintain the causal ordering. Instead, we attach to the messages the causal information that includes the messages with immediate dependency relation. This allows us to continue with the system execution without waiting for the handoff procedure to end. Therefore, the handoff management process that we propose is characterized by allowing an asynchronous transfer of the mobile hosts among the cells of the system. Moreover, this handoff management process does not interrupt the communication at any moment. Therefore, our handoff management process is asynchronous.

### Correctness Proof

To show that our algorithm ensures the causal delivery (correctness), we provide a correctness proof. In order to do the proof as simple as possible, we focus on the novel part for the wireless channels, which is the information (bits) attached to the messages and the causal information stored at the base stations. We show that with this information we ensure the causal order.

Let two messages *m_k_* = (*p_i_*, *a*, *event*, *h*(*m_k_*)) and *m_l_* = (*p_j_*, *b*, *event*, *h*(*m_l_*)), where *p_i_* and *p_j_* are the sender mobile hosts of *m_k_* and *m_l_*, respectively, *a* and *b* are the sequential ordered logical clocks for messages of *p_i_* and *p_j_* when *m_k_* and *m_l_* are sent, respectively, and finally *h*(*m_k_*) and *h*(*m_l_*) are the structures of bits when the messages *m_k_* and *m_l_* are sent, respectively.

**Theorem 1.**


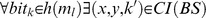
 such that 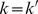, where 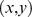 identifies a message *m_k_*, which has IDR with *m_l_*.

#### Main steps of the proof

The proof is composed by two lemmas and a proposition. The lemmas are intermediate results necessary for our proof:

- Lemma 1 shows that if *bit_k_* belongs to the causal history of a message *m_l_*, then the message identified by *bit_k_* causally precedes message *m_l_*.
- Lemma 2 indicates that the message *m_k_* has an immediate dependency relation with the other message *m_l_* if and only if the *bit_k_* belongs to the causal history of the message *m_l_*.
- Proposition 1 shows that through the bits structure (*h*(*m*)) attached to sent messages and the causal information at the base stations, we ensure the causal order (theorem 1).

**Lemma 1.**

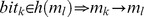

****

Proof: By Line 1, 2, we have that 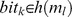 if and only if 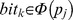 when send of *m_l_* is carried out by *p_j_*, we denote it by *send*(*p_j_, m_l_*). By using Line 2, 8, we have that 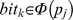 only after the delivery *m_k_* = (*p_i_*, *a*, *event*, *h*(*m_k_*)) at *p_j_*. This implies that the delivery of *m_k_* precedes the sending of *m_l_* (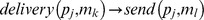). Therefore, 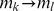.

**Lemma 2.**

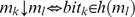

****

The proof to this lemma is divided into two steps: First, we show that 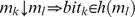 and second, we show that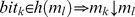.

Step 1:
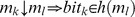



The proof is by contrapositive, we proof that 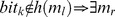
such that 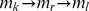; thus, the message *m_k_* has not an immediate independency relation with message *m_l_*, see Section background and definitions. We assume that 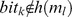. Only two events can delete *bit_k_* of *Φ*(*p_j_*) before sending *m_l_* (*send*(*p_j_, m_l_*)), these are:

- By Lines 2, 6–7, *bit_k_* is removed from *Φ*(*p_j_*) when the delivery of message *m_r_* is carried out with 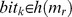 at *p_j_* (*delivery*(*p_j_, m_r_*)). Lemma 1 shows that in this case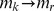. Moreover, 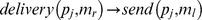 implies that 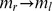 and then 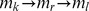. Therefore, *m_k_* does not directly precede message *m_l_*.
- By Line 1, 5, the sending of *m_r_* at *p_j_* empty *Φ*(*p_j_*). In addition, the event of *send*(*p_j_, m_r_*) takes place such that 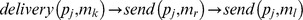. Therefore, *m_k_* does not directly precede message *m_l_*.

If neither of these two events occur, we have that 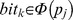 when the *send*(*p_j_,m_l_*) is carried out and by Line 1, 2, we have that 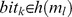.

Step 2:
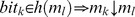



The proof is by contradiction. By lemma 1, we know that if 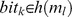 then 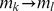 with *p_i_* ≠ *p_j_*. We suppose that there is a message *m_r_* such that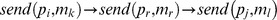, and in addition that 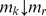. The proof considers two cases: *p_r_*≠*p_j_* and *p_r_* = *p_j_*.

- We consider the case where *p_r_*≠*p_j_* and the delivery *m_k_* causally precedes to *m_r_* (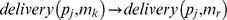) at *p_j_*. By the step 1, we know that 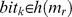. Hence, on the delivery *m_r_* (*delivery*(*p_j_, m_r_*)) at mobile host *p_j_*, *bit_k_* is deleted by Lines 2, 6–7. When performing the sending of *m_l_* (*send*(*p_j_, m_l_*)) and because of 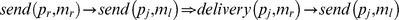, then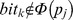 and therefore, 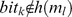, which is a contradiction.
- In the case where *p_r_* = *p_j_*, we have that 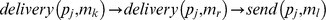, because the sending of *m_r_* (*send*(*p_j,_ m_r_*)) takes place, *bit_k_* is deleted from *Φ*(*p_j_*) by Line 1, 5 (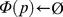). Therefore, we have that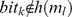, which is a contradiction.

Finally, the following proposition shows that through the bits attached to the sent messages and the causal information stored at the base stations, we ensure the causal order in the mobile distributed system.

**Proposition 1.**

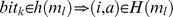

****

Proof. By Line 3, 20, we have that
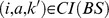
only after the delivery of message *m_k_*  = (*i*, *a*, *mes_received*(*p_i_*), *event*, *h*(*m_k_*)) at the local base station *BS.* In this case, *k*' (by line 3, 14) identifies the sent message by the base station to its local mobile hosts. In the delivery of *m_k_* at *p_j_* with *a* = *k*', we have (by Lines 2, 1–5) that *k*' =  *mes_received*(*p_j_*) and by Line 2, 8, we have that 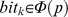. We know by lemma 2 that if 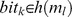
then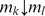. On the reception of message *m_l_* sent by *p_j_* with *m_l_* =  (*p_j_*, *b*, *mes_received*(*p_j_*), *event*, *h*(*m_l_*)) at the *BS*, by Lines 3, 7–13, we have
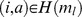
because there is in *CI*(*BS*) an element (*i*, *a*, *k*') where *k*' = *mes*_*received*(*p_j_*).

## Discussion

We compare our protocol with the related work according to four aspects: message overhead sent over wireless/wired communication channels, storage overhead, unnecessary inhibition in the message delivery, and handoff complexity (see Table 5 and 6).

**Table 5:** Causal algorithms for mobile distributed systems without unnecessary inhibition in the message delivery.

Protocol	Communication Overhead (bytes)		Types of communication	Storage overhead (bytes)	
	wireless network	wired network		Base Stations	Mobile Hosts
AV-1	0	c*n^2^ (always)	Unicast	c*(n*n^2^+n)) (always)	0
PRS	0	c*n^2^ (worst case)	Multicast	c*(n^2^+n^3^+n*s) (worst case)	0
Dependency Sequences	0	c*(s*ε) (not bounded)	Unicast	c*(n*(s*ε)) (not bounded)	0
Mobi_Causal	0	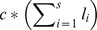 (always)	Unicast	c*(n*|*LastRcv*|) (not bounded)	0
HierarchicalClocks	0	c*s (always)	Unicast	c*(*s+φ*) (not bounded)	0
MOKA	*n/b* (worst case)	c*2n (worst case)	*Group communication* (broadcast case)	c*2n (worst case)	*n/b* (worst case)

Where *n* =  number of MHs, *s = * number of BSs, *c* is the number of bytes used to represent a integer value, *b* is the number of bits used to represent a byte, *k* is a predetermined integer parameter, *ε* is the length of the longest dependency sequence for a MH, *l_i_* represents the number of messages sent by base station BS*_i_* and for which the delivery is not yet confirmed and *LastRcv* is a control information structure that stores identifiers of messages received by a MH.

**Table 6:** Comparisons of handoff complexity among all protocols.

Protocol	Number of sent messages between *BSs* (source and target)	Message size (bytes)	Handoff management
AV-1	1	c*n^2^	Synchronous
Dependency Sequences	1	c*(s*ε) (not bounded)	
Hierarchical Clocks	1	c*s	
Mobi_Causal	1	c*n	
MOKA	1	c*n (worst case)	Asynchronous

Where *n* =  number of *MHs, s = * number of *BSs*, *c* is the number of bytes used to represent a integer value, *k* is a predetermined integer parameter, *ε* is the length of the longest dependency sequence for a *MH* and *l_i_* represents the number of messages sent by base station *BS_i_* and for which the delivery is not yet confirmed.

### Message and storage overhead and unnecessary inhibition in the message delivery

In order to reduce the overhead sent over wireless communication channels, the protocols AV-2 [13], AV-3 [13], YHH [14], LH [5], and KHC [7], ensure causal ordering at and according to the Base Stations. However, these protocols give rise to two main problems. First, the causal order seen by the MHs greatly differ from the causal orders of messages in which they were originally sent. Secondly, they introduce unnecessary inhibition at the message delivery. This unnecessary inhibition is due to the serialization of messages at the BSs level, since a base station is unable to detect mutual concurrency between messages occurring at different MHs within a single cell.

In contrast, the protocols AV-1 [13], PRS [15], Dependency sequences [4], Hierarchical clocks [4], Mobi_Causal [9], and our protocol, MOKA, maintain causal ordering explicitly among MHs. Hence, unnecessary inhibition never occurs. Nevertheless, the protocols AV-1, PRS, Dependency sequences and Mobi_Causal highly increase the messages' overhead sent over the wired communication channels and the storage overhead at base station, see Table 5.

The communication and storage overhead for these protocols is as follows. AV-1 attaches a matrix of size 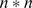 to messages sent over the wired network. Therefore, the protocol AV-1 generates a constant communication overhead over the wired network of size 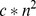 bytes where *c* is the number of bytes used to represent an integer value and *n* is the number of mobile hosts; and in order to achieve the causal ordering AV-1 needs a storage overhead of size 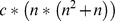 bytes. For the PRS, the communication overhead in the wired channel is dynamic having a worst case of size 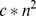
bytes. We note that, in this protocol, the updating process of the control structures considers the acknowledgement by the mobile hosts for each message received to its local base station which increases the delay in the communication. Moreover, the storage overhead of PRS in the worst case at base station is of size 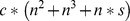 bytes where *s* is the number of base stations.

The work of Dependency Sequences attach per messages in the wired network an overhead of size 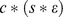 bytes, where *ε* is the length of the longest dependency sequence for a MH, see Table 5. In order to bound the size of *ε,* Praskash and Singhal [4] propose periodically using global checkpoints; however the global checkpoint is an expensive operation. In addition, the storage overhead of DS at base station is of size 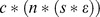 bytes, see Table 5. Next, for Mobi_causal the overhead sent over the wired communication channels is equal to 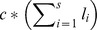
bytes, where *l_i_* represents the number of messages sent by BS*_i_* and for which the delivery is not yet confirmed. In the worst case, this number can be equal to *n*. Another drawback of Mobi_causal is the unbounded growth of control information stored (*LastRcv*) on each *BS* in order to achieve the causal ordering.

The work of Hierarchical clocks only sends overhead over the wired communication channels of size 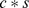
bytes; nevertheless, the identification of causal predecessors of an event involves the evaluation of a recurrence relation which imposes high communication and computation overheads. This protocol uses a hierarchical clock, *φ*, which is composed by a vector *φ^m^* and a bits vector *φ^i^* of a variable length, where the size of *φ^i^* is not bounded, see Table 5. The author as for the work of Dependency Sequences proposes the use of global checkpoints in order to bound the size of the bits vectors.

In our proposal, the MOKA protocol, the size of the control information over the wired network depends on the number of concurrent messages that immediately precede a message *m*. Since *H*(*m*) has only the most recent messages that precede a message *m*, the overhead per message in the MOKA protocol to ensure causal ordering is given by the cardinality of *H*(*m*), which can fluctuate between 0 and *n*. Therefore, the communication overhead in the wired channel is dynamic having a worst case of size 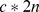
bytes. On the other hand, in our protocol, the control information attached to messages sent over the wireless network and stored at a mobile host is given by the cardinality of *h*(*m*), where *h*(*m*) is a structure of bits. Again, the size of *h*(*m*) depends on the number of concurrent messages that immediately precede to a message. Therefore, the communication overhead in the wireless channel is in the worst case of size 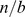 bytes, where *b* is the number of bits used to represent a byte.

On the other hand, in our protocol the storage overhead at MH is of size 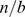 bytes and at base station is in the worst case of size 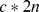
bytes. We notice that in our protocol, as for the minimal causal algorithm in [10], the likelihood that the worst case will occur approaches zero as the number of participants in the group grows. This is because the likelihood that *k* concurrent messages occur decreases inversely proportional to the size of the communication group. This behavior has been shown in [10].

### Handoff complexity

Handoff complexity indicates the amount of causal information exchanged between base stations during the handoff module execution [7], see Table 6. Here we only analyze the handoff module of the works that do not have unnecessary inhibition at the message delivery. The handoff complexity is determined by two aspects: 1) the number of sent messages between BS's and 2) the size of messages. Thus, AV-1 needs to send a message of size 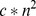
bytes when a MH moves to its new cell, see Table 6.

The handoff module proposed by the Dependency sequences, the Hierarchical clocks and the Mobi_causal needs a message of size 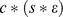, 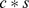
and 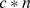 bytes, respectively, where *ε* is the length of longest dependency sequence for a MH, see Table 6. The main drawback of all these protocols is that during the handoff module execution, the other MHs cannot send messages until the handoff process concludes which inhibits the system execution and degrades the application performance.

Our protocol MOKA is the only asynchronous, and it only needs to send one message in the worst case of size 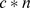 bytes. This is because the control message sent is ensured to be causally delivery along the causal messages of the MDS.

## Conclusions

The MOKA protocol has been presented. This protocol ensures the causal ordering according to the causal view of the mobile host, eliminating the inhibition effect in the message delivery. The causal protocol presented satisfies the *MDS* requirements since at the mobile hosts a low computational cost is needed because only binary operations and simple sums are used. Moreover, low memory buffer is used since only a structure of bits is stored. In addition the MOKA protocol is efficient in the overhead attached per message at the wired and the wireless communication channels. The overhead sent per message is characterized by being dynamically adapted according to the behavior of the concurrent messages. Finally, the handoff management process presented is characterized by an asynchronous execution, which allows for the transfer of a *MH* from one cell to another without the need to suspend the sending or delivery of new causal messages.
